# Clinical outcomes of transcatheter aortic valve replacement in patients with radiation-induced aortic stenosis: a systematic review and meta-analysis

**DOI:** 10.3389/fcvm.2025.1537220

**Published:** 2025-08-12

**Authors:** Daniyal Ameen, Nisarg Thakker, Rafael Contreras, Seyyed Mohammad Hashemi, Amir Nasrollahizadeh, Parsa Saberian, Dona Kuriyakose, Ehsan Amini-Salehi, Narsimha Rao Keetha, Sandeep Samethadka Nayak

**Affiliations:** ^1^ Department of Internal Medicine, Yale New Heaven Health Bridgeport Hospital, Bridgeport, CT, United States; ^2^ Cardiovascular Research Center, Hormozgan University of Medical Sciences, Bandar Abbas, Iran; ^3^ Tehran Heart Center, Cardiovascular Diseases Research Institute, Tehran University of Medical Sciences, Tehran, Iran; ^4^ St. Joseph’s Mission Hospital, Anchal, Kerala, India; ^5^ Gastrointestinal and Liver Diseases Research Center, Guilan University of Medical Sciences, Rasht, Iran; ^6^ Ohio Kidney and Hypertension Center, Middleburg Heights, OH, United States

**Keywords:** aortic stenosis, chest radiation, radiation-induced aortic stenosis, systematic review, transcatheter aortic valve replacement, transcatheter aortic valve implantation, TAVR, TAVI

## Abstract

**Background:**

Transcatheter aortic valve replacement (TAVR) is an effective treatment for severe aortic stenosis, particularly in high-risk patients unsuitable for surgical aortic valve replacement (SAVR). However, the efficacy of TAVR in patients with radiation-induced aortic stenosis remains uncertain and controversial. This meta-analysis evaluates clinical outcomes of TAVR in patients with prior chest radiation (C-XRT).

**Methods:**

A comprehensive literature search of PubMed, Scopus, and Web of Science databases was conducted through September 15, 2024. Studies comparing TAVR outcomes in patients with and without prior chest radiation were included. Statistical analysis used STATA software with a random-effects model, incorporating Knapp-Hartung correction and prediction intervals. Publication bias was assessed using funnel plots, Egger's test, Begg's test, and the trim-and-fill method.

**Results:**

The meta-analysis found no significant differences in short-term outcomes between patients with and without C-XRT. In-hospital mortality (OR: 0.81; 95% CI: 0.14–4.69), 30-day mortality (OR: 1.59; 95% CI: 0.71–3.55), and 1-year mortality (OR: 1.15; 95% CI: 0.52–2.54) were comparable. Similarly, rates of in-hospital myocardial infarction, stroke, and major bleeding showed no significant differences. The GRADE assessment indicated very low-quality evidence for most outcomes, including in-hospital mortality and stroke, and low-quality evidence for outcomes like 30-day stroke and acute kidney injury.

**Conclusion:**

TAVR appears effective in patients with prior chest radiation, with comparable short-term outcomes to non-C-XRT patients. However, due to significant heterogeneity across the included studies and the low to very low quality of evidence, these findings should be interpreted with caution. The current data remains inconclusive, and further high-quality, prospective studies with longer follow-up periods are essential to better understand the long-term risks and confirm the safety and efficacy of TAVR in this patient population.

**Systematic Review Registration:**

PROSPERO CRD42024593497.

## Introduction

Transcatheter aortic valve replacement (TAVR) has emerged as a less invasive alternative to surgical aortic valve replacement (SAVR) for patients with severe aortic stenosis, particularly in those who are considered high-risk for open-heart surgery (1–3). As the indications for TAVR expand, a diverse patient population is being evaluated for this procedure, including individuals with a history of radiation therapy (4–8). Prior radiation exposure, especially in the thoracic region is known to contribute to cardiovascular complications such as valvular heart disease, coronary artery disease, and conduction system abnormalities and is often at least a moderate contraindication for SAVR (4, 9–13).

Radiation-induced heart disease (RIHD) is a consequence of therapeutic radiation, typically manifesting years after exposure (14–16). The pathophysiology involves damage to endothelial cells, leading to fibrosis, microvascular changes, and accelerated atherosclerosis (14, 16, 17). Patients who have undergone radiation therapy for malignancies like Hodgkin's lymphoma or breast cancer are at increased risk for developing calcific aortic stenosis and other cardiac sequelae that may necessitate interventions like TAVR (16, 18, 19).

**Figure 1:**
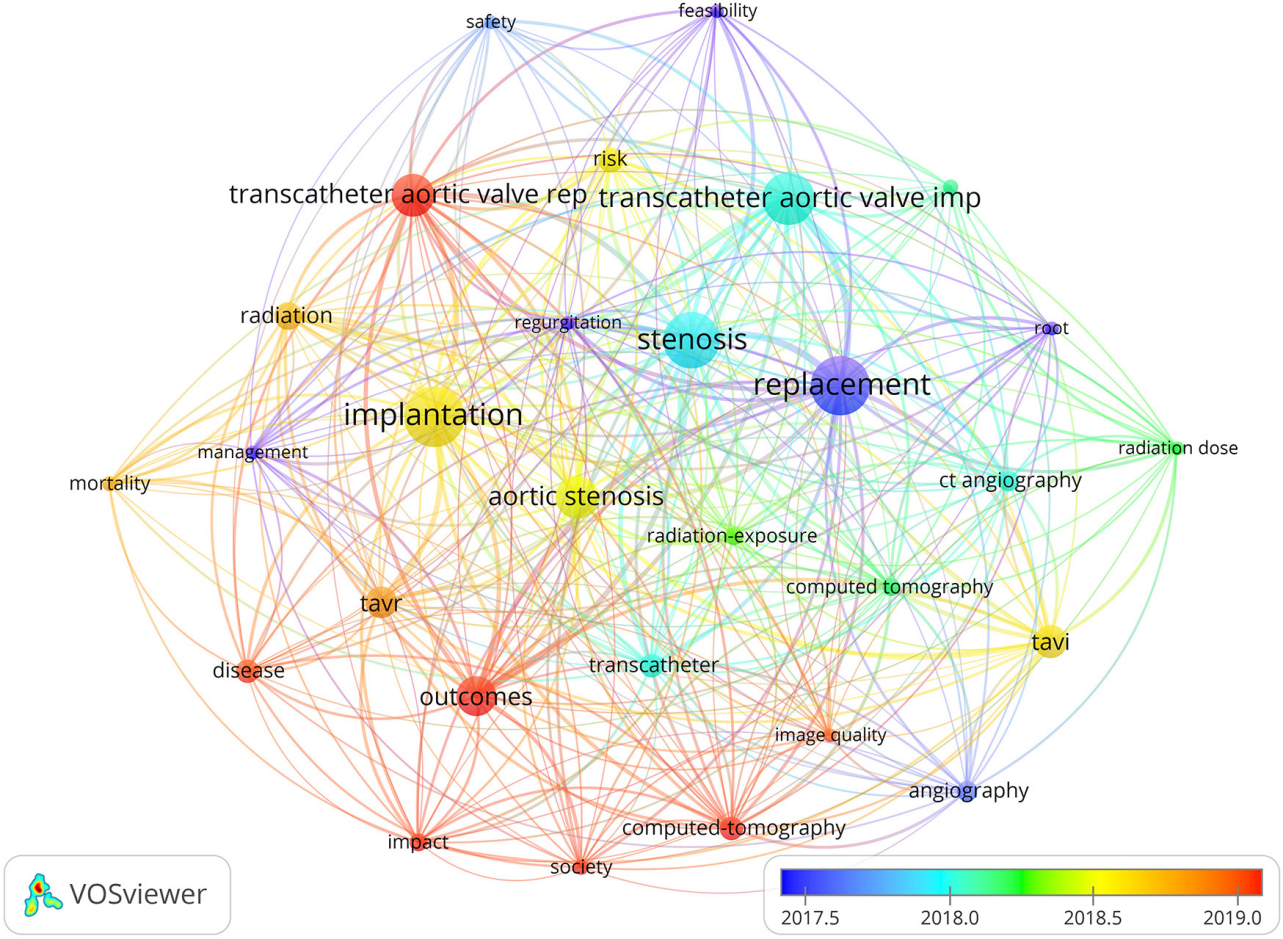
Network visualization of the landscape of research topics related to transcatheter aortic valve replacement (TAVR) and its clinical outcomes (key elements such as “TAVR,” “outcomes,” and “aortic stenosis” are prominently featured, highlighting their increasing significance in the field).

Despite the growing number of radiation-exposed patients requiring TAVR, there is limited data regarding their clinical outcomes compared to non-radiated patients. Given the paucity of comprehensive data and the clinical significance of understanding how prior radiation affects TAVR outcomes, a systematic evaluation is warranted. This meta-analysis aims to compare the clinical outcomes of patients undergoing TAVR with a history of radiation therapy to those without such exposure. By pooling data from multiple studies, we seek to provide clarity on whether prior radiation impacts procedural success, perioperative complications, and long-term survival in TAVR patients (Figure 1).

## Methods

### Setting

This meta-analysis was carried out following the guidelines set by the Preferred Reporting Items for Systematic Reviews and Meta-Analyses (PRISMA) and Cochrane Handbook for Systematic Reviews (20, 21). A thorough literature search was executed across various databases, including PubMed, Scopus and, Web of Science from their inception up to September 15, 2024. Several keywords were searched, including terms such as “Radiation Therapy,” “Radiotherapy,” “Radiation-exposed,” “Chest Radiation,” “Thoracic Radiation,” “Transcatheter Aortic Valve Replacement,” “TAVI,” and “TAVR.” No limitations were imposed regarding language or geographical location. The detailed search strategy is presented in Supplementary Table S1. The protocol of the study is registered in PROSPERO (CRD42024593497).

### Study selection

The study selection process involved two independent reviewers (PS and DA) who screened the titles and abstracts of all identified studies. Full-text articles were retrieved for further evaluation if they appeared to meet the inclusion criteria or if there was uncertainty. Discrepancies between reviewers were resolved through discussion or consultation with a third reviewer (EA-S).

### Inclusion and exclusion criteria

Studies were included if they compared clinical outcomes of patients undergoing TAVR with and without prior chest radiation therapy. Eligible studies were required to be peer-reviewed, published in scientific journals, and provide quantitative data on primary and secondary outcomes, such as mortality, stroke, myocardial infarction, bleeding, and heart failure exacerbation. Additionally, studies had to report data with adequate follow-up periods, including in-hospital, 30-day, 1-year, and, where available, follow-up periods extending beyond 1 year. Commentaries, case reports, case series, editorials, and books were excluded. Furthermore, studies that did not report sufficient data on relevant clinical outcomes were excluded.

### Quality assessment

The quality of included studies was assessed using the Joanna Briggs Institute (JBI) checklist, which is a comprehensive tool for evaluating the methodological quality of studies. The checklist includes 8 items for cross-sectional studies, 10 items for case-control studies, and 11 items for cohort studies. Each item can be answered with “yes,” “no,” “unclear,” or “not applicable” (22–24). Two independent reviewers (PS and DA) assessed each study using the JBI checklist, with disagreements resolved through consensus with a third reviewer (EA-S).

### Data extraction

Two reviewers (PS and DA) independently extracted data from the included studies. Extracted data included author names, year of publication, study design, sample size, details of radiation therapy exposure, TAVR procedural details, and clinical outcomes. For studies that did not report all necessary data, corresponding authors were contacted for additional information. Outcomes of interest included in-hospital, 30-day, and 1-year mortality, cardiovascular events (e.g., stroke, myocardial infarction), bleeding complications, heart failure exacerbation, and need for pacemaker implantation post-TAVR. Disagreements resolved through consensus with a third reviewer (EA-S).

### Statistical analysis

The statistical analysis was conducted using STATA 18. For binary outcomes, the odds ratio (OR) was reported as the effect measure. For continuous outcomes, the standardized mean difference (SMD) was chosen as the summary statistic. A leave-one-out sensitivity analysis was performed to assess the impact of each individual study on the overall effect. A random-effects model with restricted maximum likelihood estimation was selected for the analysis to account for potential variability across studies. In cases where the number of included studies was fewer than 10, the Knapp-Hartung correction was applied to adjust for the small sample size. Prediction interval analysis was also conducted to estimate the range of effects in future studies. Heterogeneity across studies was evaluated using the *I*^2^ statistic and the Cochrane *Q* test. Heterogeneity was considered significant if *I*^2^ exceeded 50% and the *P*-value was below 0.1. Publication bias was assessed using contour-enhanced funnel plots, Egger's test, Begg's test, and the trim-and-fill method (25, 26). The certainty of evidence for each outcome was assessed using the GRADE (Grading of Recommendations Assessment, Development and Evaluation) approach. Evaluations were conducted within the GRADE Pro software, which applies standardized criteria for downgrading and upgrading evidence quality. Downgrading was based on five domains: risk of bias, inconsistency, indirectness, imprecision, and publication bias. Upgrading was considered based on three domains: large magnitude of effect, plausible confounding, and evidence of a dose-response gradient. Based on these domains, the certainty of evidence was rated as high, moderate, low, or very low.

## Results

### Study selection process

**Figure 2:**
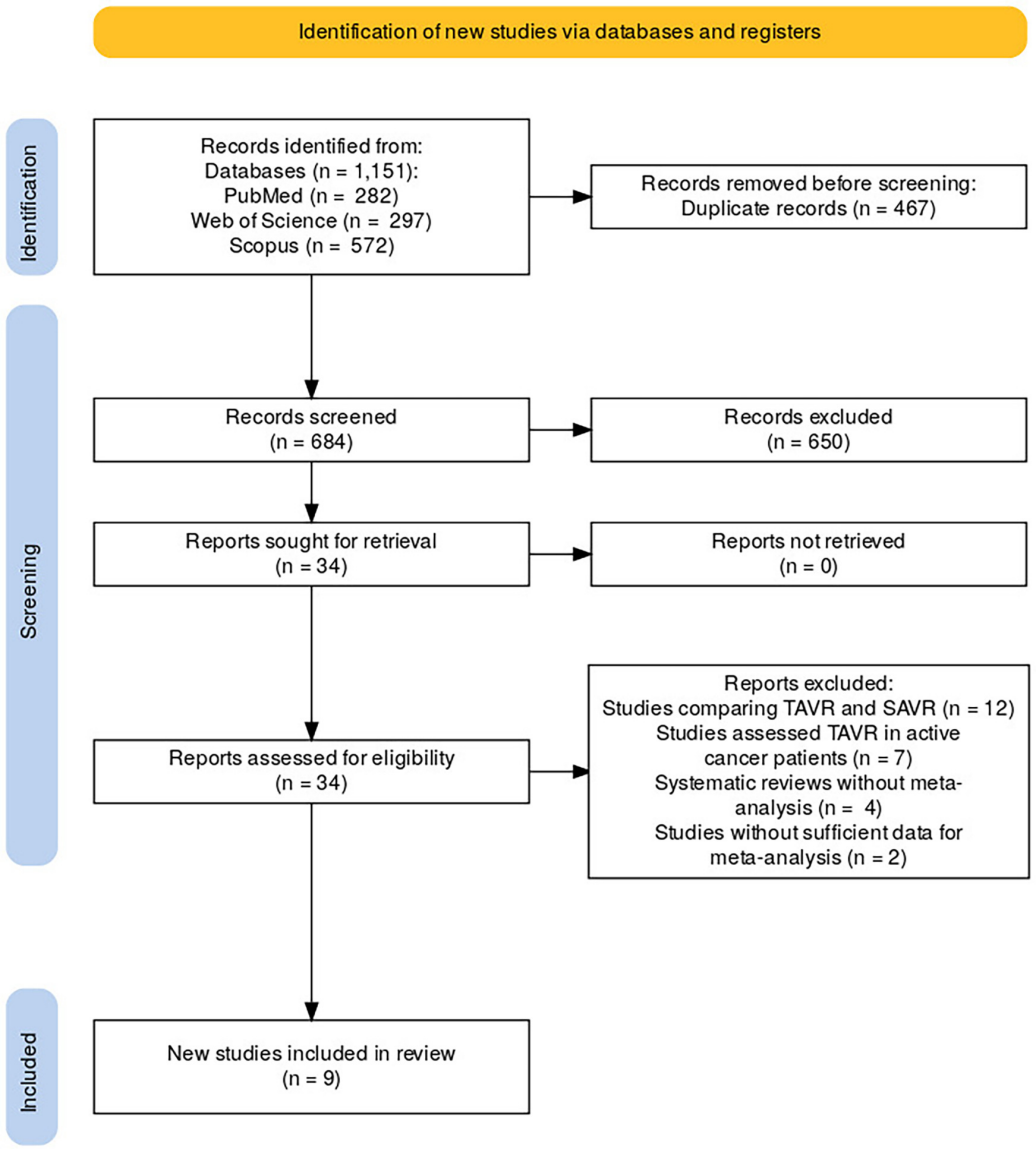
Study selection process.

A comprehensive search of databases including PubMed, Web of Science, and Scopus was conducted, yielding a total of 1,151 records (PubMed = 282, Web of Science = 297, Scopus = 572). After removing 467 duplicate records, 684 unique studies were screened for eligibility. Following the screening process, 650 records were excluded based on their relevance to the study's criteria. The full texts of 34 studies were sought for further retrieval and assessment. Upon reviewing the 34 full-text reports, 25 studies were excluded for the following reasons: 12 studies compared TAVR with Surgical Aortic Valve Replacement (SAVR), seven studies focused on patients undergoing TAVR who were actively receiving cancer treatment, four studies were systematic reviews without meta-analyses, and two studies did not provide sufficient data for inclusion in the meta-analysis. After applying these criteria, nine studies were eligible and included in the final review (Figure 2).

### Study characteristics

The included studies in this meta-analysis evaluated the clinical outcomes of patients undergoing TAVR with prior chest radiation therapy (C-XRT) compared to those without radiation exposure. Most of the studies were conducted in the United States (27–33), with two originating from France (34, 35).

The total sample sizes across the studies ranged from 52 to 296,670 patients. The number of patients with a history of chest radiation therapy ranged from 16 to 2,780 across the included studies. All the included studies were cohort studies and median follow-up periods across the studies ranged from 1 to 60 months. All the included studies had high quality (Supplementary Table S2; Table 1).

**Table 1:** Characteristics of included studies.

Study name	Year of publication	Country	Journal	Type of study	Time period of study conduction	Total sample size	Number of patients with prior radiation	Number of patients without prior radiation	Median follow-up
Agrawal et al. (27)	2019	USA	Cardio-Oncology	Cohort	2012–2017	610	75	535	17.1 months
Gajanana et al. (28)	2019	USA	Cardiovascular Revascularization Medicine	Cohort	2003–2017	1,194	44	1,150	12 months
Gajjar et al. (29)	2024	USA	Cardiovascular Revascularization Medicine	Cohort	2016–2020	296,670	515	296,155	1 months
Agrawal et al. (30)	2024	USA	The American Journal of Cardiology	Cohort	2016–2019	173,743	2,780	170,963	6 months
Kumar et al. (31)	2023	USA	Journal of Invasive Cardiology	Cohort	2012–2020	167	46	121	28 months
Boueti et al. (34)	2022	France	Heart BMJ Journal	Cohort	2006–2011	52	26	26	60 months
Dijos et al. (35)	2015	France	Open-heart BMJ journal	Cohort	2011–2013	190	16	172	6 months
Kherallah et al. (32)	2020	USA	International Journal of Cardiology	Cohort	2012–2018	150	50	100	24 months
Mohanty et al. (33)	2022	USA	Catheterization and Cardiovascular Interventions	Cohort	N/A	3,990	64	3,923	24 months

### Results of meta-analyses

#### In-hospital stay

**Figure 3:**
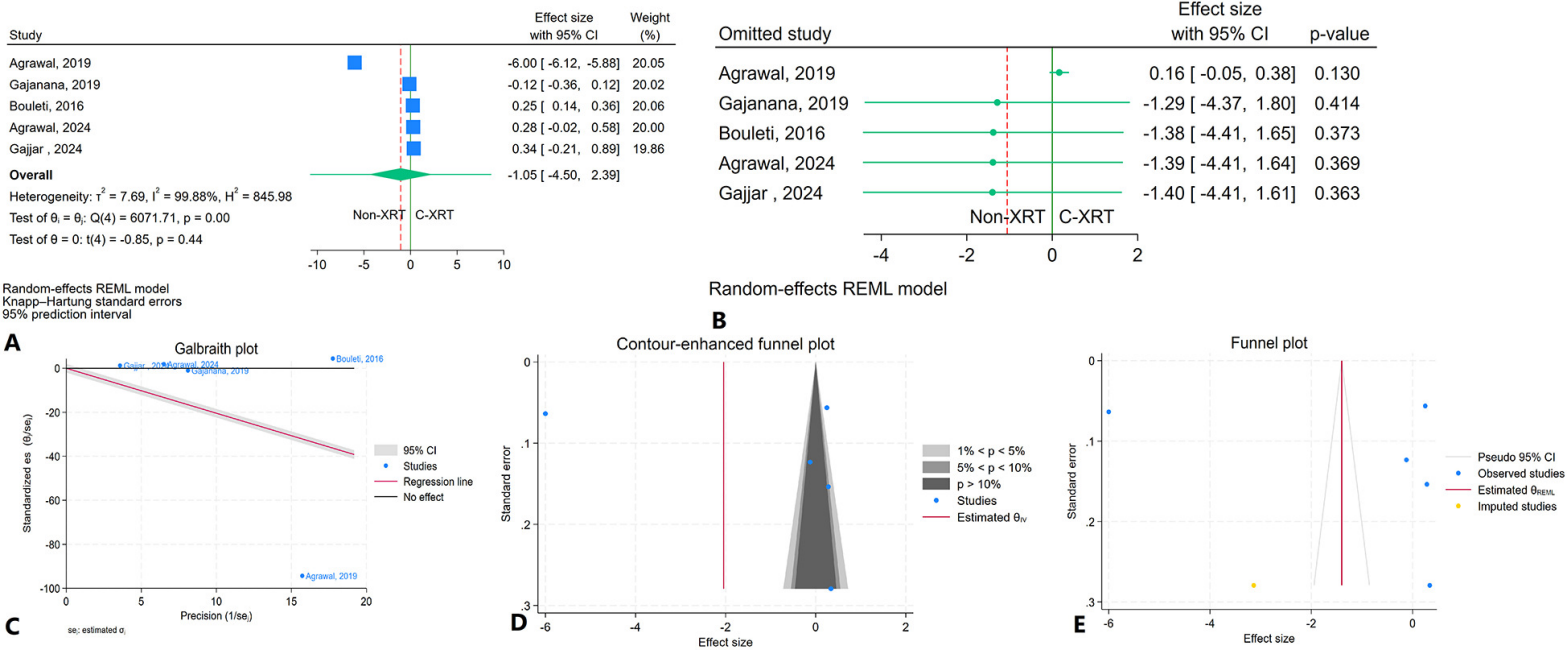
Comparison of hospital stay in patients with and without prior chest radiation therapy. **(A)** Forest plot. **(B)** Sensitivity analysis. **(C)** Galbraith plot for heterogeneity. **(D)** Contour-enhanced funnel plot. **(E)** Trim-and-fill analysis.

The meta-analysis results showed no significant difference in hospital stay between patients receiving C-XRT and those not undergoing XRT (SMD: −1.05, 95% CI: −4.50 to 2.39, *P* = 0.44) (Figure 3A). The prediction interval varied from −14.00 to 11.90 (Figure 3A). A leave-one-out sensitivity analysis demonstrated that omitting any individual study did not significantly impact the overall outcome (Figure 3B). Heterogeneity across studies was notably high (*I*^2^ = 99.88%, *H*^2^ = 845.98, *τ*^2^ = 7.69), indicating substantial variability in the results (Figure 3C). The contour-enhanced funnel plot exhibited a symmetrical distribution, suggesting no evidence of publication bias (Figure 3D). Moreover, small study effects were not significant, as indicated by Egger's regression test (*P* = 0.36) and Begg's test (*P* = 0.46). The trim-and-fill analysis, which incorporated one additional study to adjust for potential publication bias, resulted in a minor change (SMD = −1.40, 95% CI: −4.41 to 1.61) (Figure 3E).

#### ICU stay

**Figure 4:**
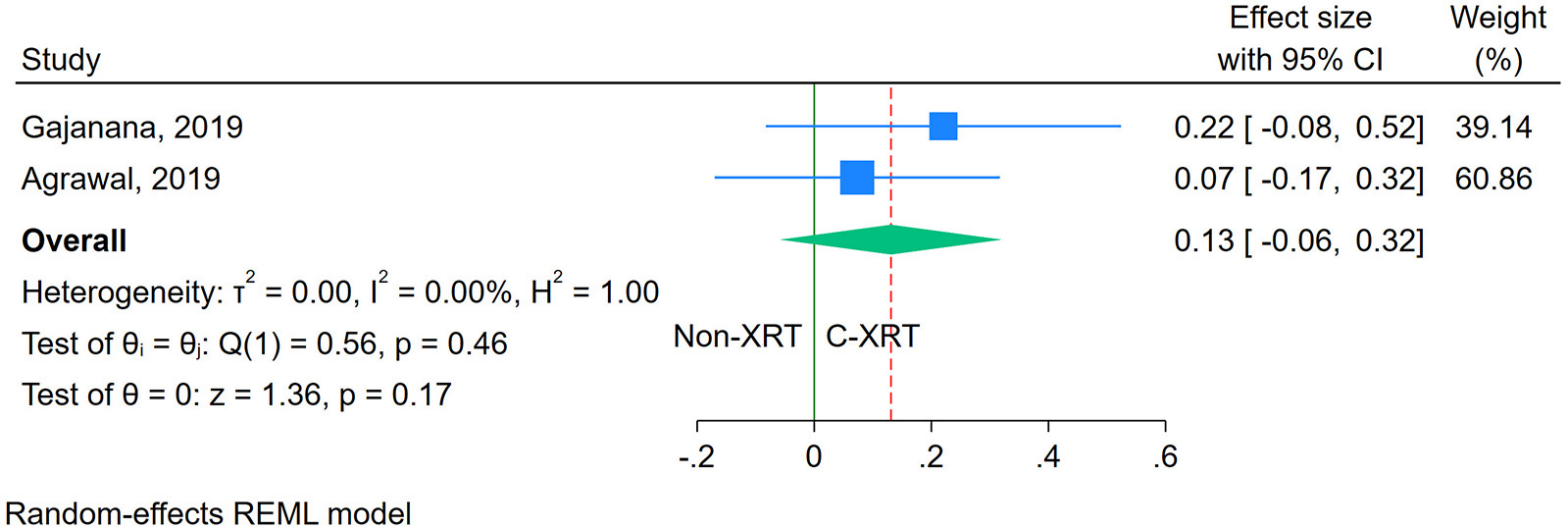
Forest plot of the comparison of ICU stay in patients with and without prior chest radiation therapy.

The meta-analysis comparing ICU stay between patients receiving C-XRT and those not undergoing XRT showed no significant difference (SMD: 0.13, 95% CI: −0.06 to 0.32, *P* = 0.17) (Figure 4). However, only two studies were available for this outcome, making it unfeasible to perform a sensitivity analysis. Additionally, the evaluation of publication bias was not feasible due to the limited number of studies.

#### In-hospital mortality

**Figure 5:**
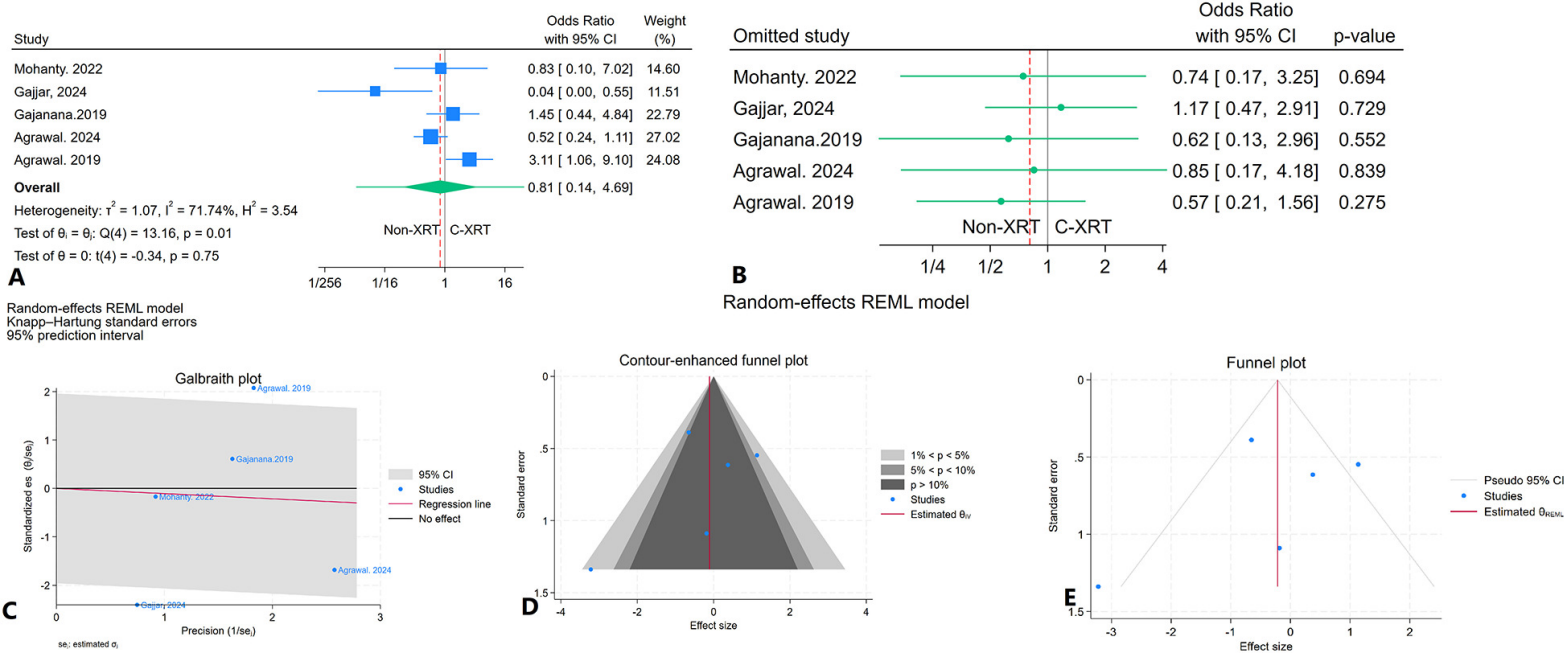
Comparison of in hospital mortality in patients with and without prior chest radiation therapy. **(A)** Forest plot. **(B)** Sensitivity analysis. **(C)** Galbraith plot for heterogeneity. **(D)** Contour-enhanced funnel plot. **(E)** Trim-and-fill analysis.

The meta-analysis comparing in-hospital mortality between patients receiving C-XRT and those not undergoing XRT showed no significant difference (OR: 0.81, 95% CI: 0.14–4.69, *P* = 0.75) (Figure 5A). The prediction interval analysis ranged from 0.01 to 34.60, indicating wide variability in potential outcomes (Figure 5A). A leave-one-out sensitivity analysis demonstrated that omitting any single study did not substantially affect the overall outcome (Figure 5B). The heterogeneity was moderate (*I*^2^ = 71.74%, *H*^2^ = 3.54, *τ*^2^ = 1.07), indicating variability across studies (Figure 5C). The contour-enhanced funnel plot showed a relatively symmetrical distribution, suggesting no significant publication bias (Figure 5D). Furthermore, Egger's regression test (*P* = 0.18) and Begg's test (*P* = 0.46) both indicated no significant publication bias. The trim-and-fill analysis did not impute any studies (Figure 5E).

### Thirty-day mortality

**Figure 6:**
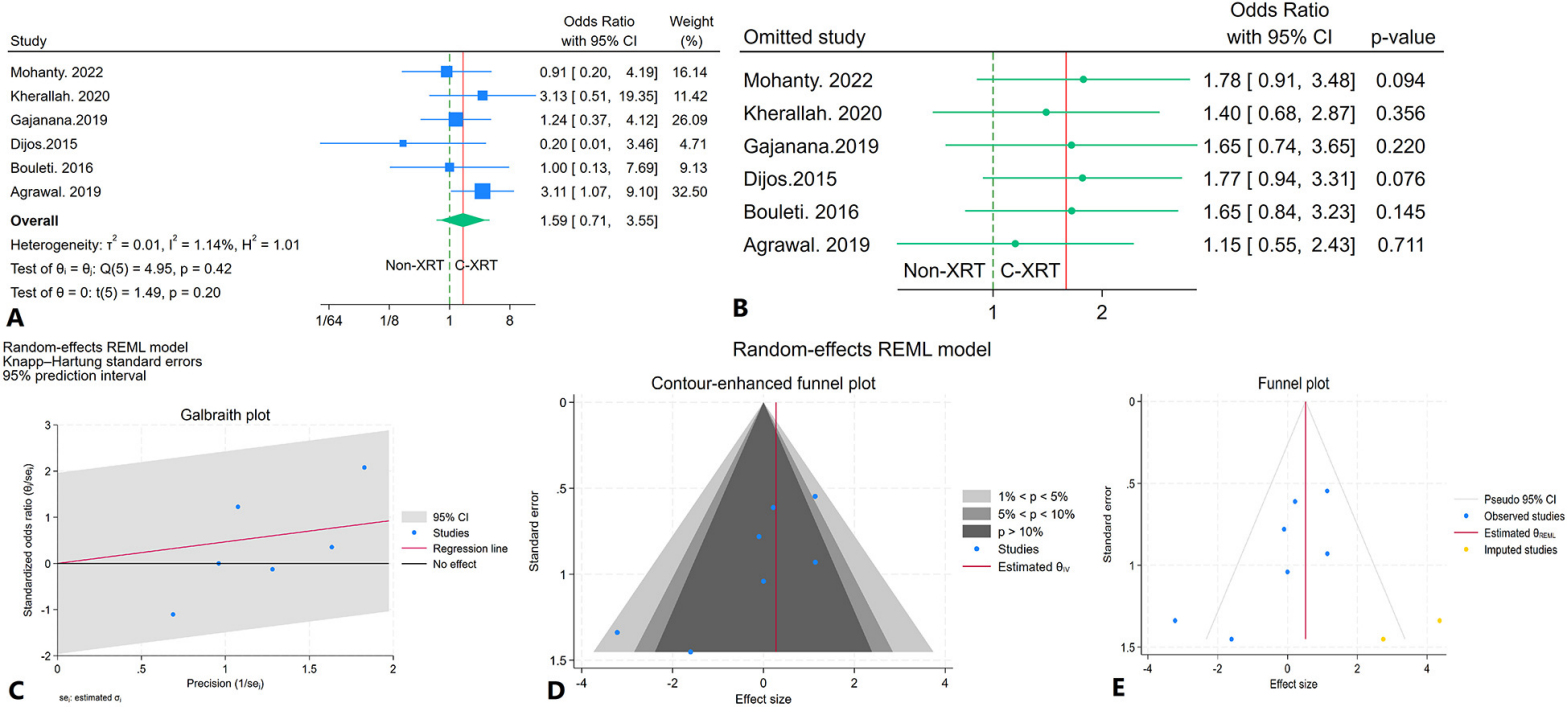
Comparison of in 30-day mortality in patients with and without prior chest radiation therapy. **(A)** Forest plot. **(B)** Sensitivity analysis. **(C)** Galbraith plot for heterogeneity. **(D)** Contour-enhanced funnel plot. **(E)** Trim-and-fill analysis.

The meta-analysis comparing 30-day mortality between patients receiving C-XRT and those not undergoing XRT showed no significant difference (OR: 1.59, 95% CI: 0.71–3.55, *P* = 0.20) (Figure 6A). The 95% prediction interval ranged from 0.09 to 11.11, indicating considerable variability in potential future outcomes (Figure 6A). A leave-one-out sensitivity analysis demonstrated that omitting any single study did not substantially alter the overall result (Figure 6B). Heterogeneity across studies was minimal (*I*^2^ = 1.14%, *H*^2^ = 1.01, *τ*^2^ = 0.01), suggesting consistent results across the studies (Figure 6C). The contour-enhanced funnel plot showed an asymmetrical pattern, suggesting potential bias (Figure 6D). Egger's regression test was significant (*P* < 0.01), and Begg's regression test showed marginal significance (*P* = 0.07). The trim-and-fill analysis, which added two imputed studies on the right side, resulted in an adjusted OR of 1.68 (95% CI: 0.50–6.56) (Figure 6E).

### One-year mortality

**Figure 7:**
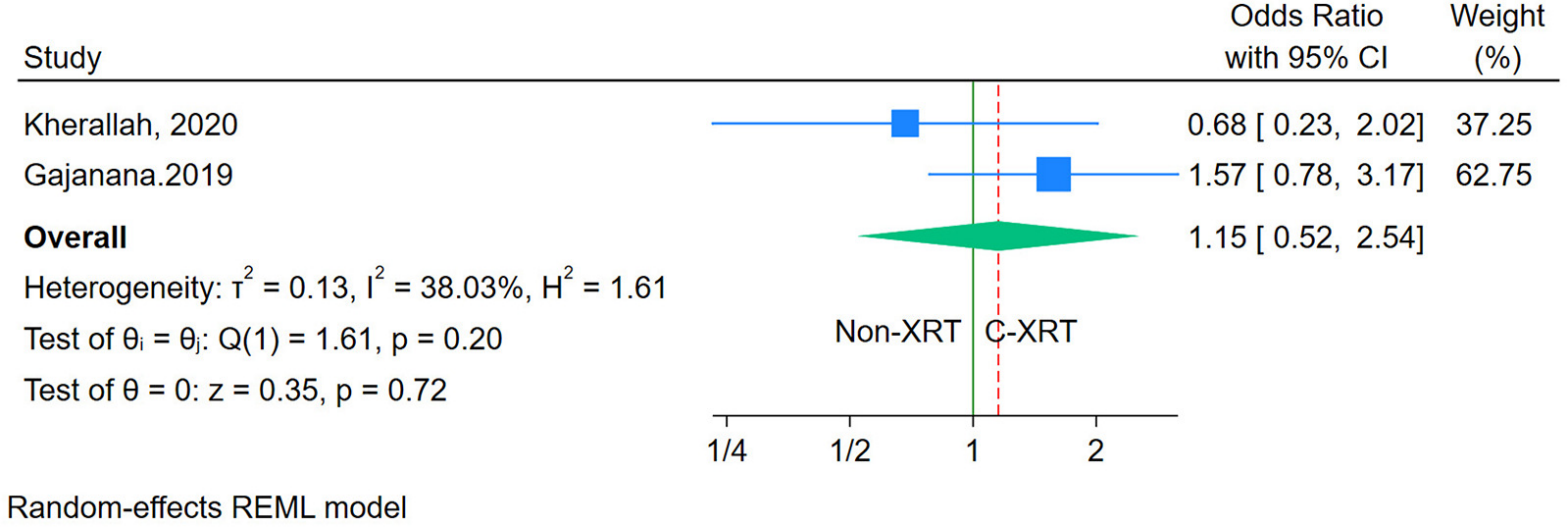
Forest plot of the comparison of 1-year mortality in patients with and without prior chest radiation therapy.

The analysis of 1-year mortality revealed no significant difference between patients receiving C-XRT and those not undergoing XRT (OR: 1.15, 95% CI: 0.52–2.54, *P* = 0.72) (Figure 7). However, since only two studies were available for this outcome, conducting a sensitivity analysis was not feasible. Furthermore, the limited number of studies also made it impossible to properly assess publication bias.

### In hospital cardiovascular mortality

**Figure 8:**
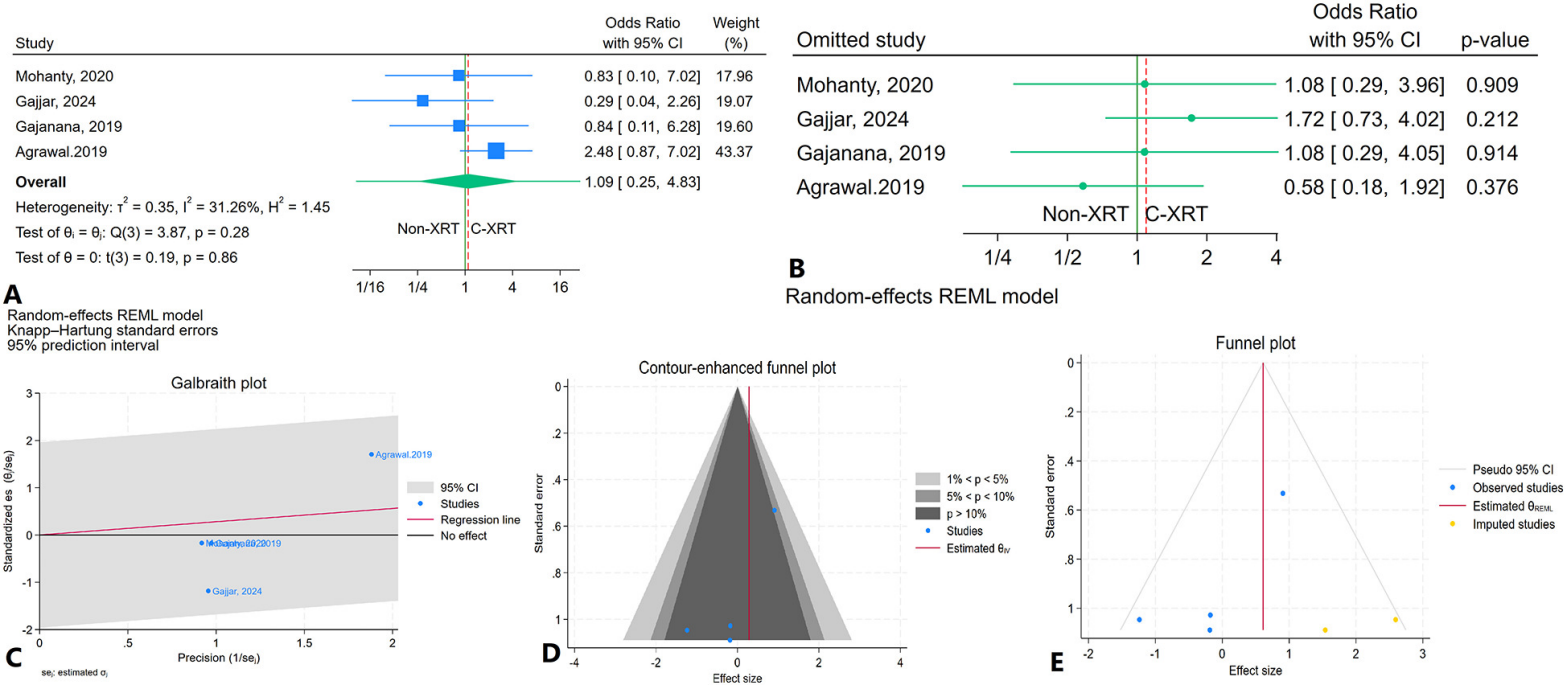
Comparison of in in hospital cardiovascular mortality in patients with and without prior chest radiation therapy. **(A)** Forest plot. **(B)** Sensitivity analysis. **(C)** Galbraith plot for heterogeneity. **(D)** Contour-enhanced funnel plot. **(E)** Trim-and-fill analysis.

The meta-analysis comparing in-hospital cardiovascular mortality between patients receiving C-XRT and those not undergoing XRT showed no significant difference (OR: 1.09, 95% CI: 0.25–4.83, *P* = 0.86) (Figure 8A). The 95% prediction interval was broad, ranging from 0.03 to 33.39, indicating considerable variability in potential future outcomes (Figure 8A). A leave-one-out sensitivity analysis demonstrated that excluding any individual study did not significantly alter the overall results (Figure 8B). Heterogeneity was not significant (*I*^2^ = 31.26%, *H*^2^ = 1.45, *τ*^2^ = 0.35) (Figure 8C). The contour-enhanced funnel plot appeared relatively asymmetrical, suggesting potential publication bias (Figure 8D). However, publication bias was significant based on Egger's test (*P* = 0.07) but not significant according to Begg's test (*P* = 0.70). The trim-and-fill analysis imputed two studies on the right side, adjusting the odds ratio to 1.84 (95% CI: 0.68–4.96) (Figure 8E).

### Thirty-day cardiovascular mortality

**Figure 9:**
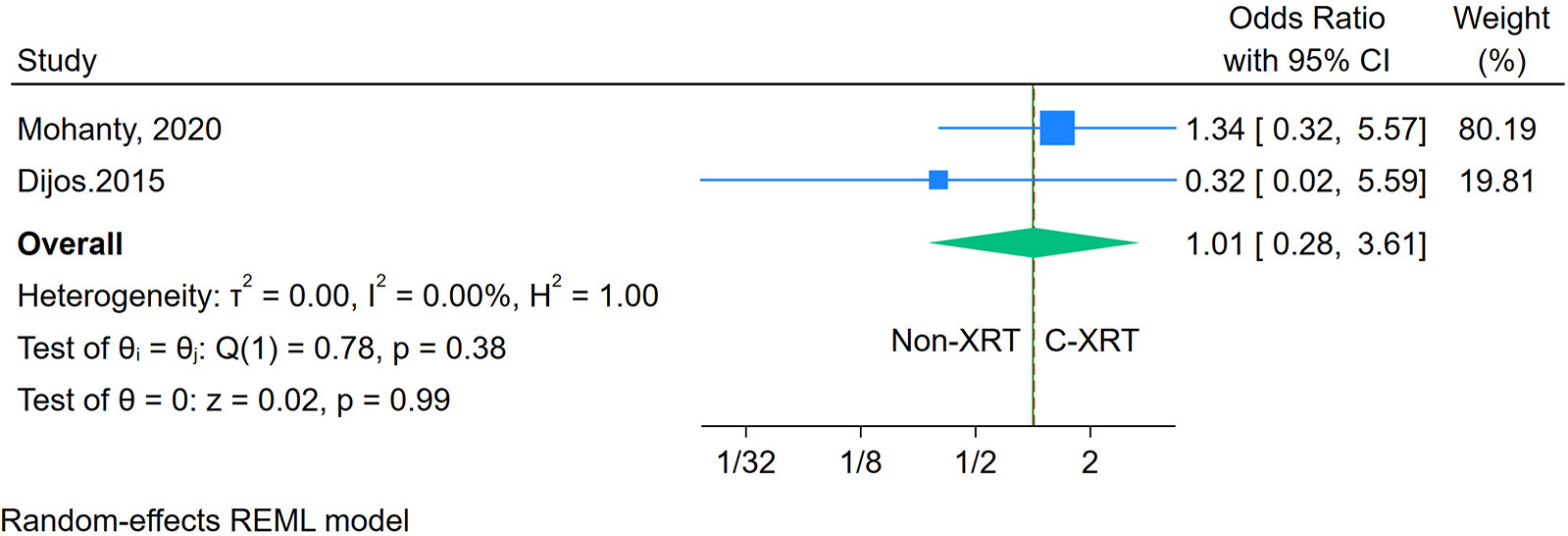
Forest plot of the comparison of 30-day cardiovascular mortality in patients with and without prior chest radiation therapy.

The meta-analysis comparing 30-day cardiovascular mortality between patients receiving C-XRT and those not undergoing XRT revealed no significant difference (OR: 1.01, 95% CI: 0.28–3.61, *P* = 0.99) (Figure 9). However, with only two studies available, it was not possible to conduct a sensitivity analysis. Moreover, the assessment of publication bias and prediction was also not feasible due to the limited number of studies.

### In hospital myocardial infarction

**Figure 10:**
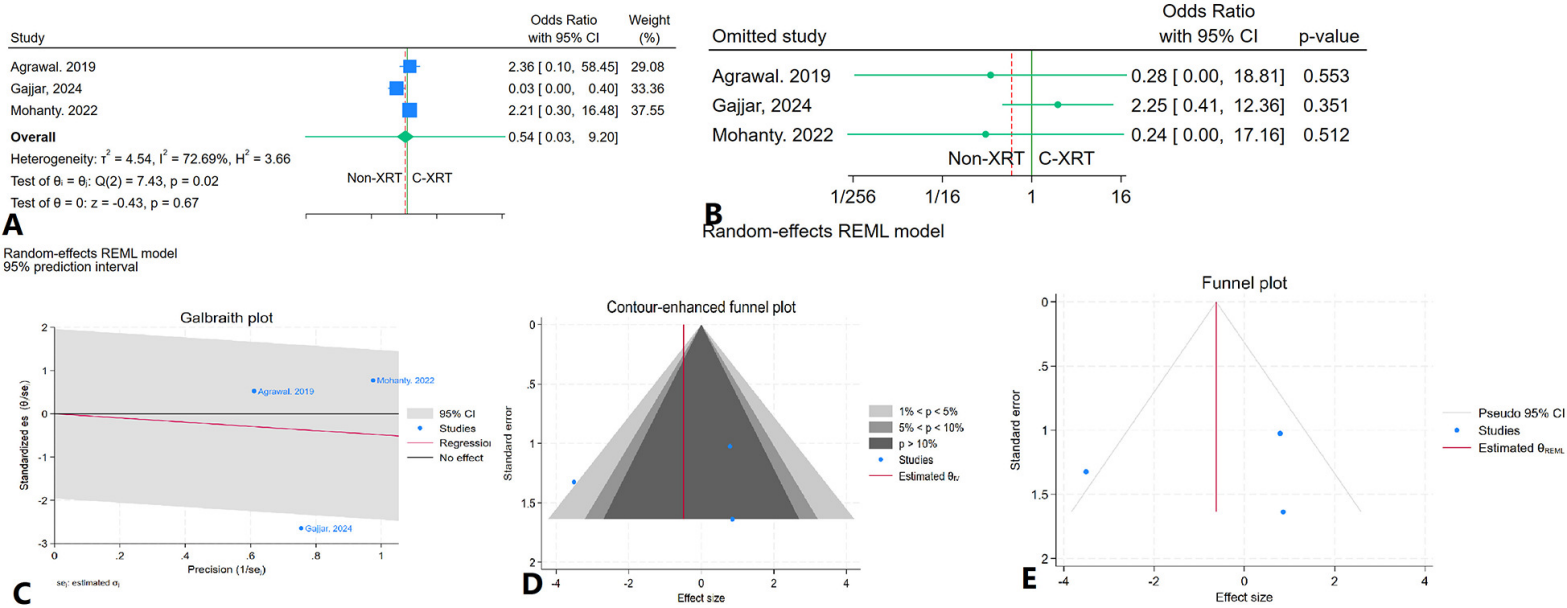
Comparison of in in hospital infarction in patients with and without prior chest radiation therapy. **(A)** Forest plot. **(B)** Sensitivity analysis. **(C)** Galbraith plot for heterogeneity. **(D)** Contour-enhanced funnel plot. **(E)** Trim-and-fill analysis.

The meta-analysis assessing in-hospital infarction between patients receiving C-XRT and those not undergoing XRT found no significant difference (OR: 0.54, 95% CI: 0.03–9.20, *P* = 0.67) (Figure 10A). A leave-one-out sensitivity analysis showed that removing any single study did not notably change the overall findings (Figure 10B). There was significant heterogeneity (*I*^2^ = 72.69%, *H*^2^ = 3.66, *τ*^2^ = 4.54), indicating substantial variability between the included studies (Figure 10C). The contour-enhanced funnel plot was fairly symmetrical, pointing to a lack of potential publication bias (Figure 10D). Publication bias was further ruled out by Egger's test (*P* = 0.99) and Begg's test (*P* = 1.00). Additionally, the trim-and-fill analysis did not add any imputed studies (Figure 10E).

### In hospital major bleeding

**Figure 11:**
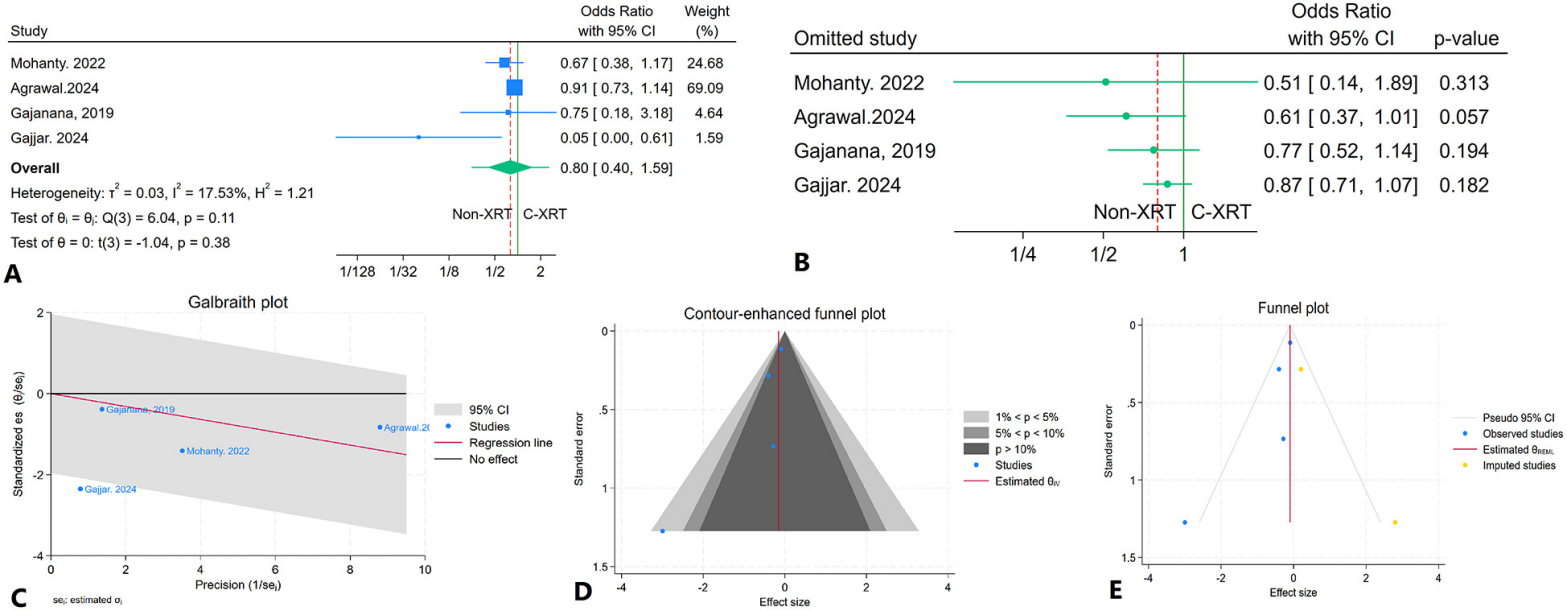
Comparison of in hospital major bleeding in patients with and without prior chest radiation therapy. **(A)** Forest plot. **(B)** Sensitivity analysis. **(C)** Galbraith plot for heterogeneity. **(D)** Contour-enhanced funnel plot. **(E)** Trim-and-fill analysis.

The meta-analysis evaluating in-hospital major bleeding incidents among patients receiving C-XRT compared to those not undergoing XRT revealed no significant difference (OR: 0.80, 95% CI: 0.40–1.59, *P* = 0.38) (Figure 11A). The prediction interval ranged from 0.30 to 2.11 (Figure 11A). A leave-one-out sensitivity analysis indicated that the exclusion of any single study did not significantly alter the overall results (Figure 11B). There was minimal heterogeneity observed (*I*^2^ = 17.53%, *H*^2^ = 1.21, *τ*^2^ = 0.03) (Figure 11C). The contour-enhanced funnel plot displayed asymmetry, suggesting potential publication bias (Figure 11D). This was further supported by Egger's test, which indicated a significant result (*P* = 0.04), while Begg's test did not show significance (*P* = 0.30). Additionally, the trim-and-fill analysis incorporated two imputed studies, resulting in an odds ratio of OR = 0.90 (95% CI: 0.74–1.09) (Figure 11E).

### Thirty-day major bleeding

**Figure 12:**
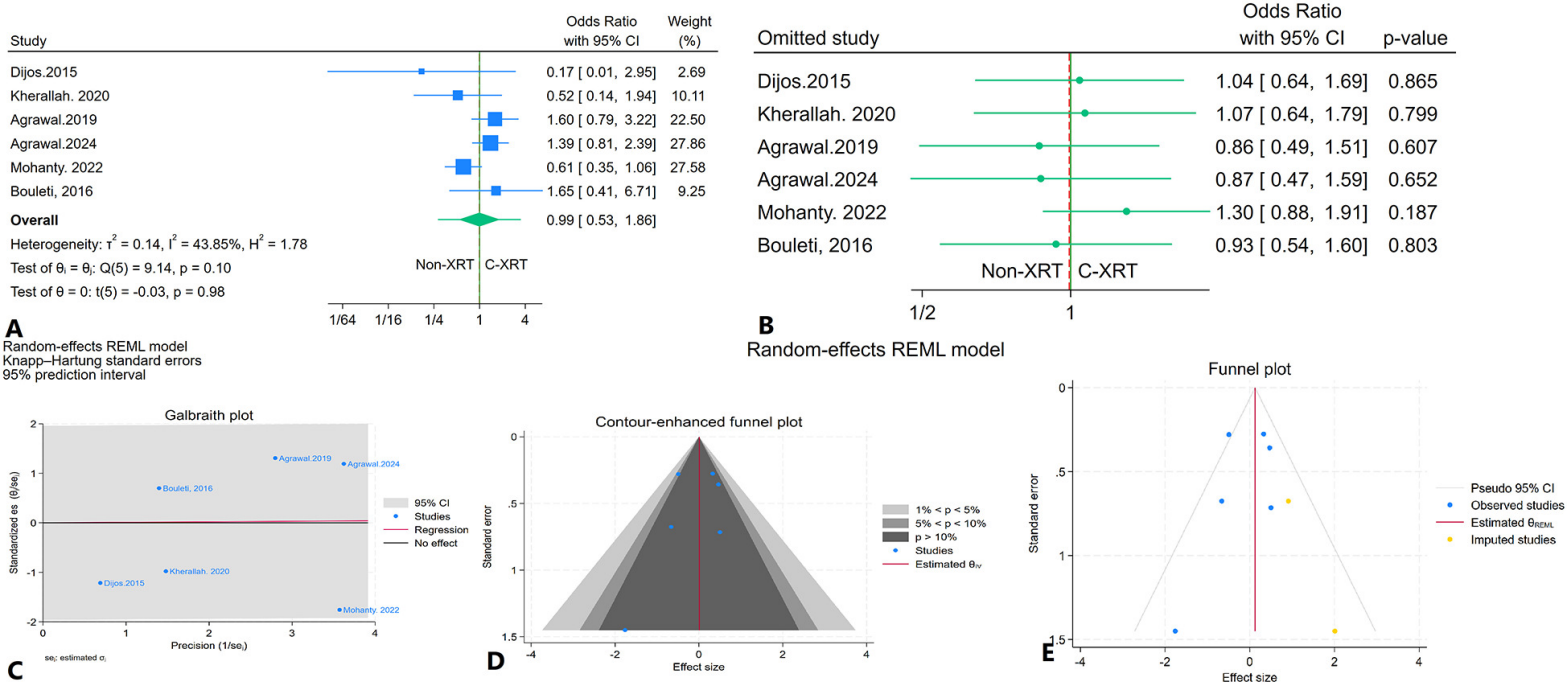
Comparison of in 30-day major bleeding in patients with and without prior chest radiation therapy. **(A)** Forest plot. **(B)** Sensitivity analysis. **(C)** Galbraith plot for heterogeneity. **(D)** Contour-enhanced funnel plot. **(E)** Trim-and-fill analysis.

The meta-analysis investigating 30-day major bleeding events in patients receiving C-XRT compared to those not undergoing XRT found no significant difference (OR: 0.99, 95% CI: 0.53–1.86, *P* = 0.98) (Figure 12A). The prediction interval spanned from 0.28 to 3.35 (Figure 12A). A leave-one-out sensitivity analysis demonstrated that the removal of any single study did not significantly affect the overall results (Figure 12B). There was no significant heterogeneity detected (*I*^2^ = 43.85%, *H*^2^ = 1.78, *τ*^2^ = 0.14) (Figure 12C). The contour-enhanced funnel plot exhibited a relatively symmetrical pattern, indicating no apparent publication bias (Figure 12D). This finding was corroborated by Egger's test, which yielded a non-significant result (*P* = 0.35), and Begg's test also showed no significance (*P* = 0.70). Furthermore, the trim-and-fill analysis added two imputed studies, resulting in an odds ratio of OR = 1.13 (95% CI: 0.71–1.79) (Figure 12E).

### In-hospital stroke

The meta-analysis evaluating in-hospital stroke rates between patients treated with C-XRT and those not undergoing XRT revealed no statistically significant difference (OR: 1.21, 95% CI: 0.11–13.20, *P* = 0.81) (Supplementary Figure S1A). The 95% prediction interval extended from 0.02 to 811.54, indicating considerable variability in potential outcomes (Supplementary Figure S1A). Sensitivity analysis, performed using the leave-one-out method, confirmed that the exclusion of any individual study did not substantially alter the overall results (Supplementary Figure S1B). However, there was considerable heterogeneity across the included studies (*I*^2^ = 85.37%, *H*^2^ = 6.84, *τ*^2^ = 1.75) (Supplementary Figure S1C). The contour-enhanced funnel plot displayed a generally symmetrical distribution (Supplementary Figure S1D). Both Egger's test (*P* = 0.87) and Begg's test (*P* = 1.00) demonstrated no significant signs of bias. Furthermore, the trim-and-fill analysis did not impute any missing studies (Supplementary Figure S1E).

### Thirty-day stroke

The meta-analysis comparing 30-day stroke incidence between patients receiving C-XRT and those not undergoing XRT showed no significant difference (OR: 0.71, 95% CI: 0.34–1.51, *P* = 0.25) (Supplementary Figure S2A). The 95% prediction interval ranged from 0.15 to 3.17 (Supplementary Figure S2A). A leave-one-out sensitivity analysis demonstrated that removing any single study did not substantially alter the overall results (Supplementary Figure S2B). There was no significant heterogeneity (*I*^2^ = 0.00%, *H*^2^ = 1.00, *τ*^2^ = 0.00) (Supplementary Figure S2C). The contour-enhanced funnel plot appeared symmetrical, implying no indication of publication bias (Supplementary Figure S2D). Publication bias was further ruled out with Egger's test (*P* = 0.81) and Begg's test (*P* = 1.00). The trim-and-fill analysis, which imputed one study on the right side, adjusted the OR to 0.71 (95% CI: 0.37–1.42) (Supplementary Figure S2E).

### AKI after TAVR

The meta-analysis that examined acute kidney injury (AKI) following the procedure in patients who received C-XRT compared to those who did not reveal no significant differences (OR: 0.42, 95% CI: 0.08–2.15, *P* = 0.22) (Supplementary Figure S3A). The 95% prediction interval ranged from 0.01 to 17.00. A sensitivity analysis revealed that after the removal of Kherallah et al. (32), patients without a history of C-XRT experienced significantly lower rates of AKI following TAVR (OR: 0.30, 95% CI: 0.11–0.81, *P* = 0.01) (Supplementary Figure S3B). The level of heterogeneity was high (*I*^2^ = 69.29%, *H*^2^ = 3.26, *τ*^2^ = 1.03 (Supplementary Figure S3C). The contour-enhanced funnel plot was symmetrical, suggesting minimal publication bias (Supplementary Figure S3D). Additionally, both Egger's test (*P* = 0.22) and Begg's test (*P* = 0.46) supported the absence of significant publication bias. The trim and fill analysis did not insert any study (Supplementary Figure S3E).

### HF exacerbation after TAVR

The meta-analysis assessing heart failure exacerbation between patients receiving C-XRT and those not undergoing XRT found no significant overall difference (OR: 1.49, 95% CI: 0.96–2.33, *P* = 0.07) (Supplementary Figure S4A). The 95% prediction interval ranged from 0.64 to 3.46 leave-one-out sensitivity analysis demonstrated that removing any single study did not substantially alter the overall results (Supplementary Figure S4B). Heterogeneity was low (*I*^2^ = 27.42%, *H*^2^ = 1.38, *τ*^2^ = 0.07), (Supplementary Figure S4C). The contour-enhanced funnel plot showed a symmetrical pattern, indicating no significant evidence of publication bias (Supplementary Figure S4D). Both Egger's test (*P* = 0.48) and Begg's test (*P* = 0.54) also showed no significant publication bias. Furthermore, the trim-and-fill analysis did not add any imputed studies (Supplementary Figure S4E).

### PPM implantation after TAVR

The meta-analysis evaluating PPM implantation after TAVR between patients receiving C-XRT and those not undergoing XRT found no significant overall difference (OR: 1.46, 95% CI: 0.70–3.05, *P* = 0.26). The 95% prediction interval ranged from 0.38 to 5.49 (Supplementary Figure S5A). leave-one-out sensitivity analysis demonstrated that removing any single study did not substantially alter the overall results (OR: 1.83, 95% CI: 1.27–2.61, *P* < 0.01) (Supplementary Figure S5B). Heterogeneity was not significant (*I*^2^ = 44.84%, *H*^2^ = 1.81, *τ*^2^ = 0.20) (Supplementary Figure S5C). The contour-enhanced funnel plot displayed an asymmetrical pattern, suggesting potential publication bias (Supplementary Figure S5D). Both Egger's test (*P* < 0.01) and Begg's test (*P* = 0.07) further confirmed the presence of significant publication bias. Moreover, the trim-and-fill analysis, which added two imputed studies, adjusted the OR to 1.98 (95% CI: 0.92–3.87) (Supplementary Figure S5E).

### Results of GRADE assessment

In the meta-analysis, the GRADE assessment revealed that the evidence quality for several outcomes was predominantly low or very low. Specifically, in-hospital stay, in-hospital mortality, 30-day mortality, in-hospital cardiovascular mortality, in-hospital myocardial infarction, in-hospital major bleeding, PPM after TAVR, and in-hospital stroke were all assigned a very low grade. Conversely, 30-day major bleeding, 30-day stroke, AKI after TAVR and, HF exacerbation after TAVR, were graded as having low evidence quality (Table 2).

**Table 2:** Summary-of-findings (SoF) table of GRADE assessment.

Quality assessment							Quality
No of studies	Design	Risk of bias	Inconsistency	Indirectness	Imprecision	Other considerations	
In-hospital stay							
5	Observational studies	No serious risk of bias	Very serious	No serious indirectness	No serious imprecision	None	⊕〇〇〇 VERY LOW
In-hospital mortality							
5	Observational studies	No serious risk of bias	Serious	No serious indirectness	No serious imprecision	None	⊕〇〇〇 VERY LOW
30-day mortality							
6	Observational studies	No serious risk of bias	No serious inconsistency	No serious indirectness	No serious imprecision	Reporting bias	⊕〇〇〇 VERY LOW
In hospital cardiovascular mortality							
4	Observational studies	No serious risk of bias	No serious inconsistency	No serious indirectness	No serious imprecision	Reporting bias	⊕〇〇〇 VERY LOW
In hospital myocardial infarction							
3	Observational studies	No serious risk of bias	Serious	No serious indirectness	No serious imprecision	None	⊕〇〇〇 VERY LOW
In hospital major bleeding							
4	Observational studies	No serious risk of bias	Serious	No serious indirectness	No serious imprecision	Reporting bias	⊕〇〇〇 VERY LOW
30-day major bleeding							
6	Observational studies	No serious risk of bias	No serious inconsistency	No serious indirectness	No serious imprecision	None	⊕⊕〇〇 LOW
In-hospital stroke							
4	Observational studies	No serious risk of bias	Very serious	No serious indirectness	No serious imprecision	None	⊕〇〇〇 VERY LOW
30-day stroke							
4	Observational studies	No serious risk of bias	No serious inconsistency	No serious indirectness	No serious imprecision	None	⊕⊕〇〇 LOW
AKI after TAVR							
5	Observational studies	No serious risk of bias	Serious	No serious indirectness	No serious imprecision	Strong association	⊕⊕〇〇 LOW
HF exacerbation after TAVR							
7	Observational studies	No serious risk of bias	No serious inconsistency	No serious indirectness	No serious imprecision	None	⊕⊕〇〇 LOW
PPM after TAVR							
7	Observational studies	No serious risk of bias	No serious inconsistency	No serious indirectness	No serious imprecision	Reporting bias	⊕〇〇〇 VERY LOW

## Discussion

The adverse events of chest radiation therapy C-XRT in cancer survivors regarding the cardiovascular system encompass a wide spectrum including myocardial and pericardial damage, vascular heart disease, conduction system abnormality, and valvulopathy. Aortic stenosis (AS) is the most common valvular heart disease regarding C-XRT (36, 37). Previous studies have declared the superiority of TAVR to SAVR in high-risk patients with a history of C-XRT (5, 38, 39). Despite the superiority of TAVR to SAVR in patients with C-XRT, there is limited data regarding TAVR key outcomes between patients with prior C-XRT and those without.

In this comprehensive meta-analysis comparing outcomes of TAVR in patients with prior C-XRT vs. those without, we found no significant differences in key short-term clinical outcomes, including mortality, cardiovascular events, and procedural complications. This challenges earlier concerns regarding the elevated risks of TAVR in patients with radiation-induced heart disease, while also providing a broader analysis across multiple clinical endpoints.

One of the notable findings of our study is that patients with XRT undergoing TAVR experienced outcomes comparable to those without C-XRT in the short term. This was reflected in key perioperative metrics, such as in-hospital and 30-day mortality, MI, bleeding, and stroke. Additionally, in-hospital and ICU stay were similar between the two groups. These findings suggest that TAVR can be considered in patients with prior radiation therapy, without an increased risk of immediate adverse events. This result is crucial for clinical decision-making, as it reinforces TAVR as a preferred treatment strategy for high-risk C-XRT patients, particularly when SAVR is challenging due to frailty or other comorbidities.

Our study extended the analysis by incorporating data from studies with longer follow-up periods, ranging up to 60 months. While short-term outcomes were largely similar between C-XRT and non C-XRT groups, subtle trends emerged in studies with extended follow-up. Although, our meta-analysis did not show statistically significant differences in 1-year mortality, radiation-induced cardiovascular damage may take years to manifest fully, leading to calcification, and fibrosis, resulting in late-onset complications such as valve degeneration, HF, and increased cardiovascular mortality which warrants close clinical surveillance (40). Although overall meta-analysis demonstrated no significant difference regarding HF exacerbation and PPM implantation, Ganjana et al. study with 1 year follow-up period, reported a significantly lower incidence of HF exacerbation in non C-XRT patients (28). Additionally, two studies by Kherallah et al. and Agrawal et al. both following patients for more than 1 year, demonstrated a higher prevalence of PPM implantation in patients with a history of C-XRT (27, 32). Regarding long-term survival, Bouleti et al. had the longest follow-up period among the included studies for 5 years and reported similar 5-year survival between the two groups (34). These findings highlight the importance of long-term monitoring in C-XRT patients after TAVR.

Prior meta-analysis has examined the outcomes of TAVR in patients with a history of C-XRT, though our findings extend and refine this body of work. The meta-analysis by Zafar et al. explored similar outcomes in patients undergoing TAVR in short-term, reporting no significant difference in early mortality, stroke, major bleeding, and PPM implantation but observed higher rates of complications such as heart failure exacerbations and also all-cause mortality at 1-year follow-up (18). Their analysis, however, was limited to only 4 studies with shorter follow-up durations (mean: 14 months) and fewer patients with prior radiation therapy, potentially underestimating late-term risks.

### Strength and limitations

Our meta-analysis includes data from a wide range of follow-up durations, from as short as 1 month to as long as 60 months to identify the absence of significant differences in short-term outcomes while highlighting the potential for increased long-term risks. The inclusion of long-term follow-up periods adds depth to our understanding of the effects of radiation therapy on TAVR outcomes, providing new insights that were previously underreported. Furthermore, it integrates data from a broader pool of patients, including several large cohorts such as Gajjar et al. and Agrawal et al. (29, 30) with a larger number of patients undergoing TAVR in both the radiation-exposed and non-exposed groups, we provide a more statistically powerful comparison, minimizing bias that might arise from smaller studies or limited patient populations. This enhances the generalizability of our findings to the broader patient population undergoing TAVR. The use of sensitivity analyses, prediction intervals, and publication bias assessments (e.g., Egger's and Begg's tests) throughout the meta-analysis ensures the robustness of our results. In addition, we conducted leave-one-out sensitivity analyses to verify the stability of our findings, and the consistent results across studies reinforce the reliability of our conclusions. These rigorous methods provide confidence that our findings are not skewed by small-study effects or publication bias.

Despite the mentioned strength and novelty of our study, there are some limitations: first, there was significant heterogeneity across the included studies, driven by a variety of factors. One major source of heterogeneity stems from the observational study design and various follow-up durations across the included. The second possible cause of heterogeneity originates from different radiation therapy dosages received by patients (15, 41) and studies included in our analysis did not always provide detailed information on the specific dosages, making it difficult to account for this variation. Third, there was variability in the timeframe between radiation therapy and TAVR across the included studies. Some patients had undergone radiation decades before TAVR, while others received treatment more recently. This variability may influence the extent of radiation-induced cardiovascular damage and could have impacted the outcomes. Fourth, the indications for radiation therapy varied widely, with some patients receiving treatment for breast cancer, while others were treated for lymphoma or lung cancer. These differences in underlying cancer types alongside possible adjuvant therapies besides radiation, such as chemotherapy can also have cardiovascular effects. These concomitant treatments were not uniformly reported or controlled for included studies, making it challenging to isolate the impact of radiation therapy on TAVR outcomes. Finally, there was a lack of data regarding the long-term durability of surgical aortic valves in patients with prior chest radiation. Future studies comparing long-term structural valve deterioration between TAVR and SAVR in this specific population are warranted. Additionally, although we used several statistical tools to decrease bias, such as sensitivity analyses, prediction intervals, and publication bias assessments (e.g., Egger's and Begg's tests), we did not employ bootstrapping of the data. Bootstrapping could have improved the confidence intervals (CIs) and provided a more robust estimate, especially given the high variability in the data. We acknowledge that incorporating bootstrapping in future studies could further enhance the precision of the results and minimize bias due to variability.

## Conclusion

The comparable short-term outcomes between C-XRT and non-C-XRT patients found in our study are relevant for clinical practice. However, due to significant heterogeneity and the low to very low quality of evidence in the available studies, these findings should be interpreted with caution. While our results suggest that TAVR can be a feasible and safe intervention for patients with prior chest radiation, the current data remain inconclusive regarding its long-term outcomes. Our study supports TAVR as a viable option for high-risk patients who might otherwise be deemed inoperable, but the evidence is not definitive, and further high-quality, prospective studies with longer follow-up periods are essential to confirm these findings and to better assess potential late-onset risks.

## Data Availability

The original contributions presented in the study are included in the article/Supplementary Material, further inquiries can be directed to the corresponding author.
