# Supplementary material for: Clinical outcomes of transcatheter aortic valve replacement in patients with radiation-induced aortic stenosis: a systematic review and meta-analysis
Source: Front Cardiovasc Med. 2025 Aug 12;12:1537220. doi: 10.3389/fcvm.2025.1537220 (PMC12378315; doi:10.3389/fcvm.2025.1537220)
Supplement: Supplementary file 1 [file Table1.docx]

Table S1: Search formula for each database

| Database | Search formula |
| --- | --- |
| ISI | (("Radiation" OR "Radiation Therapy" OR "Radiotherapy" OR "Radiation-treated" OR "Radiation-exposed" OR "Chest Radiation" OR "Thoracic Radiation" OR "Mediastinal Radiation" OR "Radiation-Induced" OR "Post-Radiotherapy" OR "Radiation Oncology" OR "Radiation Effects" ) AND ( "Transcatheter Aortic Valve Replacement" OR "TAVR" OR "Transcatheter Aortic Valve Implantation" OR "TAVI" )) |
| PubMed | (("Radiation"[All Fields] OR "Radiation Therapy"[All Fields] OR "Radiotherapy"[All Fields] OR "Radiation-treated"[All Fields] OR "Radiation-exposed"[All Fields] OR "Chest Radiation"[All Fields] OR "Thoracic Radiation"[All Fields] OR "Mediastinal Radiation"[All Fields] OR "Radiation-Induced"[All Fields] OR "Post-Radiotherapy"[All Fields] OR "Radiation Oncology"[All Fields] OR "Radiation Effects"[All Fields])) AND (("Transcatheter Aortic Valve Replacement"[All Fields] OR "TAVR"[All Fields] OR "Transcatheter Aortic Valve Implantation"[All Fields] OR "TAVI"[All Fields])) |
| Scopus | TITLE-ABS-KEY (“Radiation" OR "Radiation Therapy" OR "Radiotherapy" OR "Radiation-treated" OR "Radiation-exposed" OR "Chest Radiation" OR "Thoracic Radiation" OR "Mediastinal Radiation" OR "Radiation-Induced" OR "Post-Radiotherapy" OR "Radiation Oncology" OR "Radiation Effects") AND TITLE-ABS-KEY ( "Transcatheter Aortic Valve Replacement" OR "TAVR" OR "Transcatheter Aortic Valve Implantation" OR "TAVI |


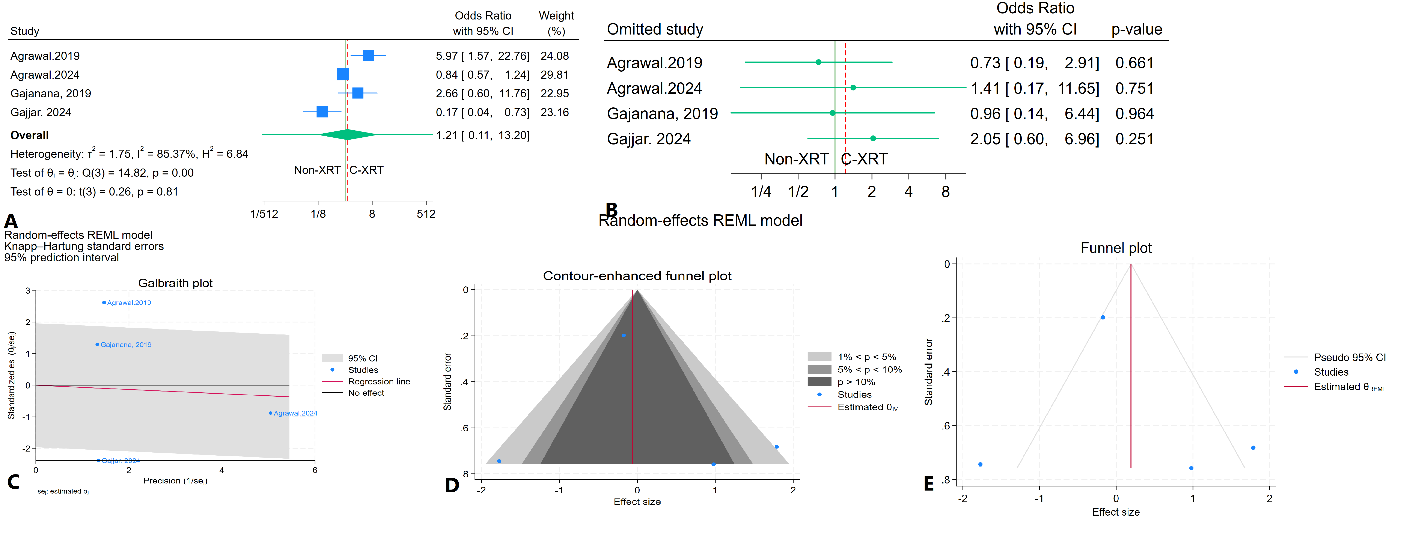


**Figure** S1**:** Comparison of in hospital stroke in patients with and without prior chest radiation therapy. **A:** Forest plot. **B:** Sensitivity analysis. **C:** Galbraith plot for heterogeneity. **D:** Contour-enhanced funnel plot. **E:** Trim-and-fill analysis.


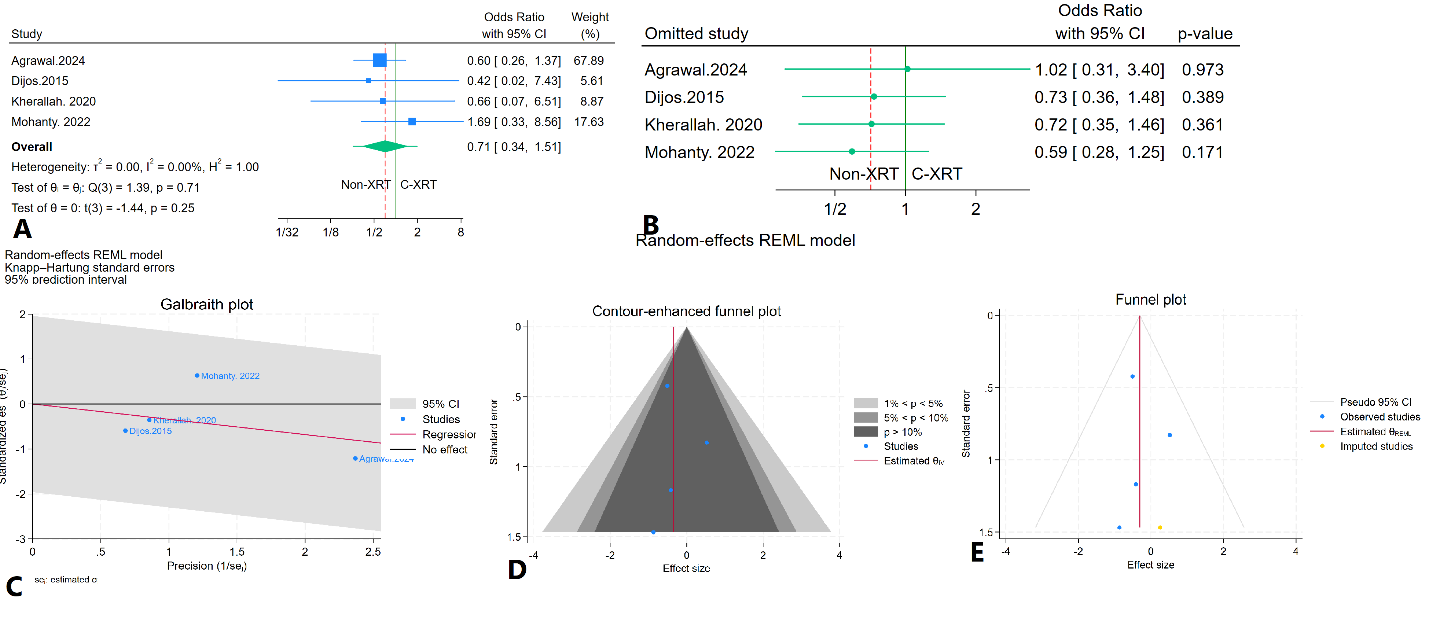


**Figure** S2**:** Comparison of 30 day stroke in patients with and without prior chest radiation therapy. **A:** Forest plot. **B:** Sensitivity analysis. **C:** Galbraith plot for heterogeneity. **D:** Contour-enhanced funnel plot. **E:** Trim-and-fill analysis.


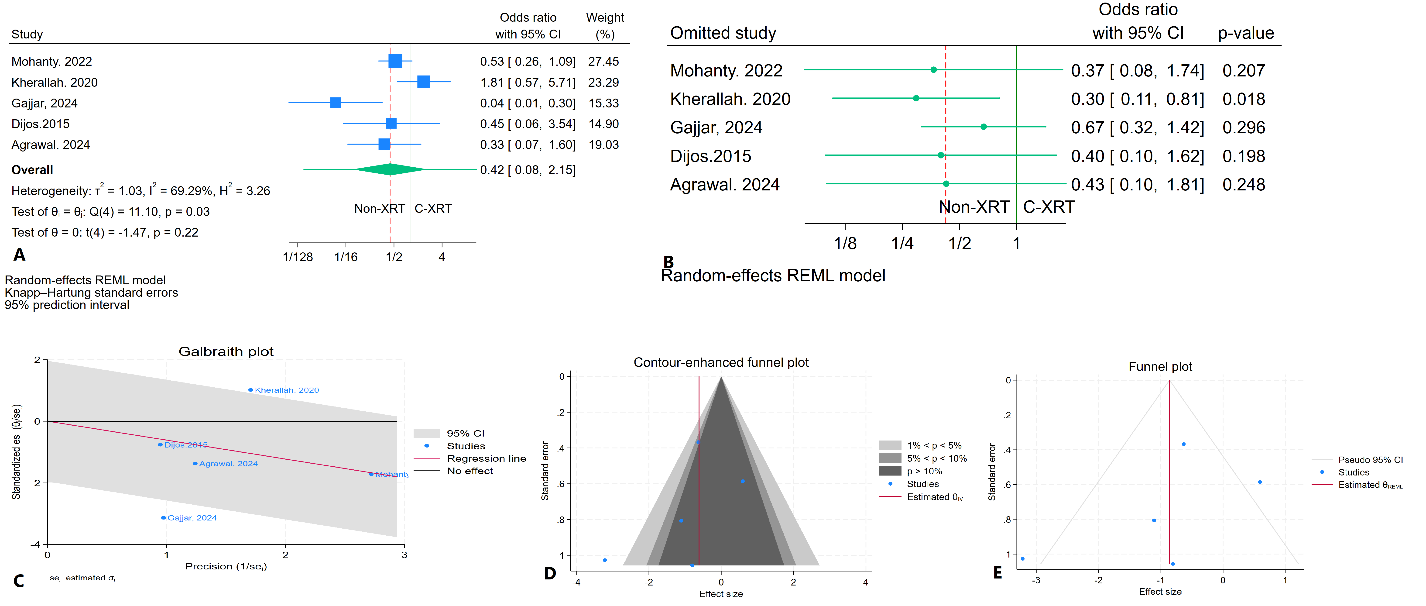


**Figure S3:** Comparison of acute kidney injury after transcatheter aortic valve replacement in patients with and without prior chest radiation therapy. **A:** Forest plot. **B:** Sensitivity analysis. **C:** Galbraith plot for heterogeneity. **D:** Contour-enhanced funnel plot. **E:** Trim-and-fill analysis.


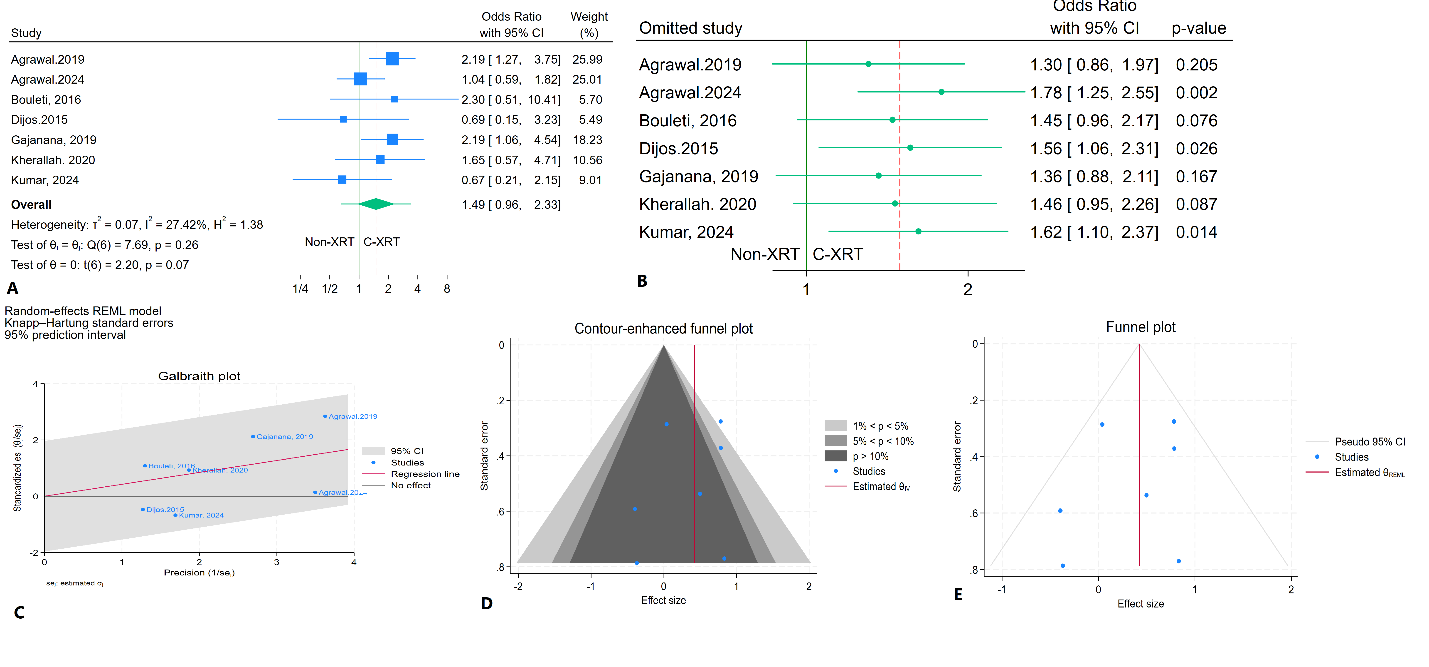


**Figure** S4**:** Comparison of heart failure exacerbation after transcatheter aortic valve replacement in patients with and without prior chest radiation therapy. **A:** Forest plot. **B:** Sensitivity analysis. **C:** Galbraith plot for heterogeneity. **D:** Contour-enhanced funnel plot. **E:** Trim-and-fill analysis.

**
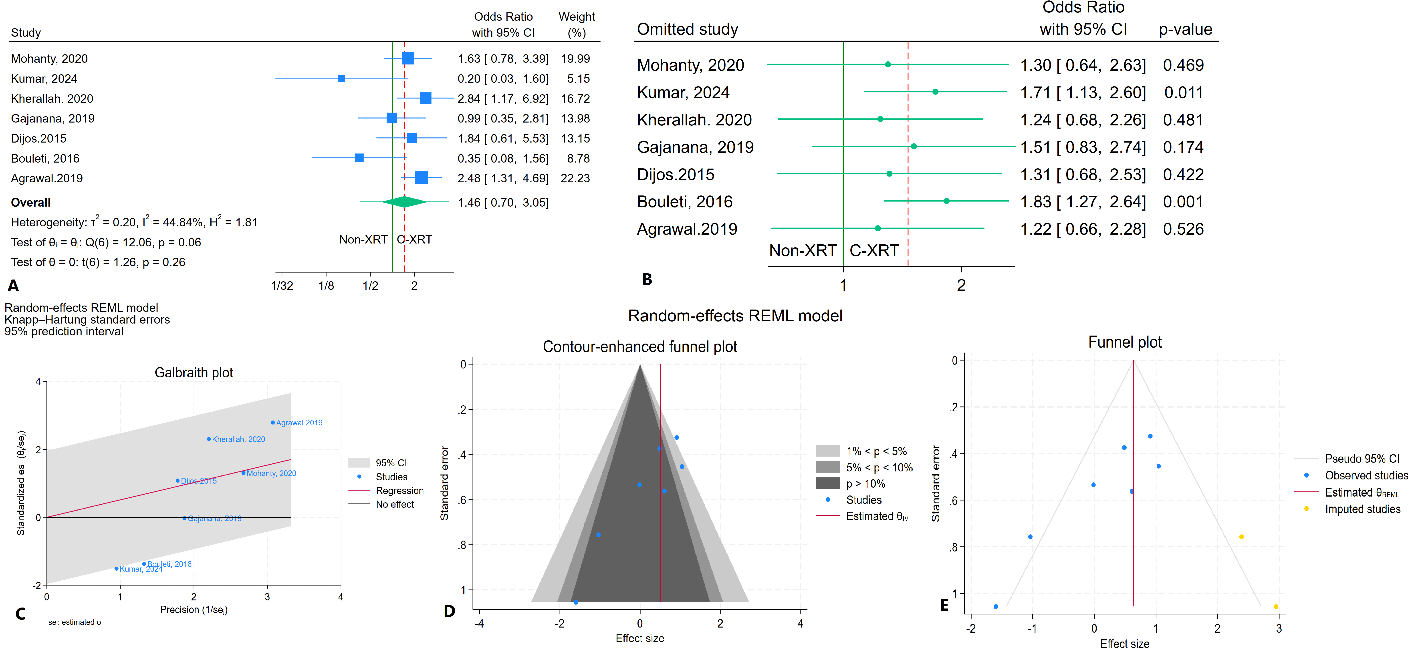
**

**Figure** S5**:** Comparison of pace maker implantation after transcatheter aortic valve replacement in patients with and without prior chest radiation therapy. **A:** Forest plot. **B:** Sensitivity analysis. **C:** Galbraith plot for heterogeneity. **D:** Contour-enhanced funnel plot. **E:** Trim-and-fill analysis.

| **Number of studies** | **Author, year** | **Question 1** | **Question 2** | **Question 3** | **Question 4** | **Question 5** | **Question 6** | **Question 7** | **Question 8** | **Question 9** | **Question 10** | **Question 11** | **Total score** |
| --- | --- | --- | --- | --- | --- | --- | --- | --- | --- | --- | --- | --- | --- |
| Study 1 | Gajjar,2024 | yes | yes | yes | yes | yes | Yes | yes | yes | unclear | no | yes | 9/11 |
| Study 2 | Agarwal,2024 | yes | yes | yes | yes | yes | Yes | yes | unclear | no | no | yes | 8/11 |
| Study 3 | Kumar, 2023 | yes | yes | yes | yes | yes | yes | yes | yes | yes | unclear | yes | 10/11 |
| Study 4 | Agarwal, 2019 | yes | yes | yes | yes | yes | Yes | yes | yes | unclear | yes | yes | 10/11 |
| Study 5 | Boueti,2022 | yes | yes | yes | yes | yes | yes | yes | yes | yes | unclear | yes | 10/11 |
| Study 6 | Dijos,2015 | yes | yes | yes | yes | yes | yes | yes | no | yes | no | yes | 9/11 |
| Study 7 | Gajanana, 2015 | yes | yes | yes | yes | yes | yes | yes | yes | yes | no | yes | 10/11 |
| Study 8 | Kherallah,2020 | yes | yes | yes | yes | yes | yes | yes | yes | yes | no | yes | 10/11 |
| Study 9 | Mohanty,2022 | yes | yes | yes | yes | yes | yes | yes | no | no | unclear | yes | 8/11 |

**Table S2:** Quality assessment of included studies
